# Global gene expression patterns in the post-pneumonectomy lung of adult mice

**DOI:** 10.1186/1465-9921-10-92

**Published:** 2009-10-05

**Authors:** Julia A Paxson, Christopher D Parkin, Lakshmanan K Iyer, Melissa R Mazan, Edward P Ingenito, Andrew M Hoffman

**Affiliations:** 1 Department of Clinical Sciences, Lung Function Testing Laboratory, Cummings School of Veterinary Medicine, Tufts University, 200 Westboro Road, North Grafton MA USA; 2 Center for Neuroscience Research, Tufts University School of Medicine, Boston, MA USA; 3 Brigham and Woman's Hospital, Harvard Medical School, Boston, MA USA

## Abstract

**Background:**

Adult mice have a remarkable capacity to regenerate functional alveoli following either lung resection or injury that exceeds the regenerative capacity observed in larger adult mammals. The molecular basis for this unique capability in mice is largely unknown. We examined the transcriptomic responses to single lung pneumonectomy in adult mice in order to elucidate prospective molecular signaling mechanisms used in this species during lung regeneration.

**Methods:**

Unilateral left pneumonectomy or sham thoracotomy was performed under general anesthesia (n = 8 mice per group for each of the four time points). Total RNA was isolated from the remaining lung tissue at four time points post-surgery (6 hours, 1 day, 3 days, 7 days) and analyzed using microarray technology.

**Results:**

The observed transcriptomic patterns revealed mesenchymal cell signaling, including up-regulation of genes previously associated with activated fibroblasts (Tnfrsf12a, Tnc, Eln, Col3A1), as well as modulation of Igf1-mediated signaling. The data set also revealed early down-regulation of pro-inflammatory cytokine transcripts and up-regulation of genes involved in T cell development/function, but few similarities to transcriptomic patterns observed during embryonic or post-natal lung development. Immunohistochemical analysis suggests that early fibroblast but not myofibroblast proliferation is important during lung regeneration and may explain the preponderance of mesenchymal-associated genes that are over-expressed in this model. This again appears to differ from embryonic alveologenesis.

**Conclusion:**

These data suggest that modulation of mesenchymal cell transcriptome patterns and proliferation of S100A4 positive mesenchymal cells, as well as modulation of pro-inflammatory transcriptome patterns, are important during post-pneumonectomy lung regeneration in adult mice.

## Background

Pulmonary emphysema is an example of a chronic disease with parenchymal destruction, where repair is relatively ineffectual [1]. To provide effective therapies for treating this disease, a better understanding of the cellular and molecular processes that govern the phenomenon of lung regeneration, and in particular alveolar regeneration, is crucial. An important approach is the analysis of tissues from animal species that retain a high degree of regenerative capacity. Adult mice are capable of regenerating functional alveoli following either lung resection or injury to a greater degree than the regenerative capacities observed in larger adult mammals [2,3]. In healthy adult mice (or rats) unilateral pneumonectomy evokes compensatory lung regeneration from the remaining lung lobes [4], in part through neoalveolarization within the existing parenchyma [5]. This regenerative process is characterized by restoration of lung volume, surface area, morphometry, DNA and protein content within 14 days, as demonstrated by our lab as well as others [6,7]. Despite a plethora of macrophysiologic and morphometric studies on lung regeneration in rodents and larger animals [2], the cellular and molecular mechanisms that regulate this process are not well understood. Previous studies using gene expression have focused on specific pathways [8] rather than global transcriptomic approaches. For example, using a gene array (588 genes) designed for analysis of transcription factors, Landenberg *et al *demonstrated a 2-fold or greater up-regulation of six genes, including early-growth response gene-1 (Egr-1), Nurr77, tristetraprolin, the inhibitor of kB-alpha (IkB-alpha), Klf-4 (GKLF) and LRG-21, all within two hours of pneumonectomy in mice [9]. The authors concluded that expression of early transcription factors (i.e. early immediate genes) activated by mechanical stress trigger a cascade of growth signals that promote lung regeneration. Likewise, repeated over-inflation of the murine lung soon after pneumonectomy (30 min) was associated with over-expression of the proto-oncogenes *c-fos *and *junB *[10], underscoring the ability of pneumonectomy-induced mechanical stretch to evoke transcription factors. Indeed, lung stretch by mechanical ventilation without pneumonectomy induces similar early immediate gene transcription [11].

While past studies have revealed early immediate genes that participate in the activation of lung regeneration, the majority of the regenerative process takes place over a prolonged period (7-14 days). Gene expression patterns corresponding to important biologic processes such as cellular proliferation, matrix formation, angiogenesis, and progenitor cell differentiation have not been fully characterized. It is also unclear from past studies why processes such as matrix formation and angiogenesis occur during the remodeling process, but are not associated with fibrosis in this context.

The objective of this study was to measure the effects of pneumonectomy (vs. sham surgery) on gene transcriptome patterns that are robustly expressed (fold change ≥1.5, or ≤ -1.5) at multiple time points during lung regeneration. Therefore, we analyzed the transcriptome (over 39,000 genes) from mouse lung tissues following unilateral pneumonectomy using Affymetrix GeneChip microarray technology. Samples were taken at four time points (6 hours, 1 day, 3 days and 7 days) post-pneumonectomy spanning the period during which the bulk (>80%) of lung regeneration occurs, as measured by changes in vital capacity [12]. Following analysis of the transcriptomic patterns, an immunohistochemical study of the regenerating parenchyma using the fibroblast markers S100A4 and αSMA was also performed at two points during lung regeneration (3 days and 7 days) to further elucidate the role of fibroblasts in this process.

## Methods

### Animals used for microarray analysis and q RT-PCR

Mice used in this study were adult (10-12 week) female C57BL/6 (20-25 g) obtained from Jackson Laboratories. All experiments were performed in accordance with NIH guidelines, as dictated by Institutional Animal Care and Use Committee at Tufts University. For each of the four time points, the mice were divided into two groups: (1) pneumonectomy (PNY) and (2) sham operated (SHAM - thoracotomy without lung resection), with eight animals in each group. Mice were anesthetized by intraperitoneal injection of ketamine (50-75 mg/kg) and xylazine (5 mg/kg), and then received 2 ml of warmed normal saline and 100 mg/kg sodium ampicillin subcutaneously. Orotracheal intubation was performed under direct visualization using a 20-gauge catheter (BD Insyte catheter; Becton, Dickinson and Co, Franklin Lakes NJ) over a flexible stylet. Mice were secured in supine position, and mechanically ventilated (AUT6110, Buxco Electronics, Wilmington, NC) at 200 tidal breaths of 0.3 ml of room air per minute, at positive end-expiratory pressure of 3 cm H_2_O during surgery and recovery.

### Pneumonectomy procedure

After achieving adequate anesthetic depth (absence of response to toe-pinch) the left thoracic wall was clipped and disinfected. The skin, chest wall and pleura were incised at the 5^th ^intercostal space, and the left lung was gently lifted through a ~5-7 mm incision and ligated at the hilum with 4-0 silk (Sofsilk, Synture Norwalk Ct). The lungs were then inflated to 30 cmH_2_0 airway pressure, and the chest wall closed during this inflation with a single interrupted suture. The skin was closed with 5-0 PDS in a simple interrupted pattern. Mice were extubated at the onset of vigorous spontaneous breathing. The mice recovered from surgery in a warmed cage, and post-operative pain was managed with buprenorphine subcutaneously (0.05 mg/kg) as soon as mice showed conscious motor control, and every 12 hours thereafter as needed (<3 days). Chow, nutrient gel (on the cage floor), and water were provided *ad libitum*. Sham pneumonectomy animals underwent an identical procedure, except that after the thoracotomy, the chest was left open for 5 minutes to simulate the conditions of the pneumonectomy group without removal of the left lung, then closed as described.

### Tissue preparation and RNA isolation

The mice were anesthetized as above at 6 hours, 1 day, 3 days and 7 days after surgery (PNY or SHAM) then euthanized by cervical dislocation. The pulmonary vasculature was perfused with ice cold Hanks balanced salt solution, the trachea cannulated, and the lungs removed *en bloc*. RNA preservation was achieved by flooding the lung intratracheally with RNAlater solution (Qiagen #76104), followed by storage of lung tissue samples in RNAlater at -80°C.

### RNA isolation and microarray analysis

Equal amounts of lung tissue were pooled from eight animals in each group (PNY or SHAM at each of 4 time points) to minimize biological variability [13]. Total RNA was prepared from the dissected lung tissue using the Qiagen RNAeasy mini kit (Qiagen #74104) according to the manufacturer's directions. The total RNA samples from the primary purification were purified a second time on Qiagen RNAeasy columns according to the manufacturer's instructions. Total RNA concentrations, A260/A280 and A260/A230 ratios were determined using a NanoDrop ND1000 spectrophotometer. All microarray analysis was performed as described in the Affymetrix GeneChip Expression Analysis Technical Manual using Affymetrix Mouse Genome 430 2.0 GeneChips and the One-Cycle cDNA Synthesis and HT IVT Labeling kits (Affymetrix Inc.). The complete microarray dataset is available (accession number GSE15999 at: http://www.ncbi.nlm.nih.gov/geo/query/acc.cgi?acc=GSE15999).

### Microarray data analysis on PNY versus SHAM animals (time-independent)

The initial goal in the analysis was to identify genes differentially regulated in the comparison between pneumonectomized and sham-operated animals. By treating all time points as replicates within their respective groups two datasets were created (PNY and SHAM), each with an n = 4. The corresponding Affymetrix CEL files were background corrected, summarized and quantile-normalized using the RMA library in BioConductor http://www.bioconductor.org, yielding one expression value per probe set for each of the 8 arrays [14]. Based on the 'Rank Products' algorithm proposed by Brietling, et. al [15], the RankProd library was employed to find differentially expressed genes. This algorithm works by performing comprehensive pair-wise comparisons to calculate a rank statistic RP_g_, defined as the probability of seeing the observed, pair-wise expression patterns for any given gene g. As a vehicle for measuring statistical significance a non-parametric *P*-value is also calculated, using 1000 permutations to determine how often the calculated RP_g _statistic would occur by chance alone. Finally, the RankProd library compares average expression between the two groups to derive a fold-change value. Genes with a reported *P*-value < 0.001 and a fold-change ≥ 1.5 or ≤ -1.5 were selected for further investigation.

### Microarray data analysis to identify temporal changes in lung regeneration (time dependent)

In a second analysis, the focus was shifted to temporal changes in gene expression during lung regeneration as opposed to overall transcriptomic patterns. With only one array per experimental condition at each time point, derivation of statistical measures and the subsequent search for truly differentially expressed genes can be challenging. However, the S-Score algorithm described by Zhang et al. (2002) and Kerns et al. (2003) provides a method for determining statistical significance when biological replicates are not available by applying pair-wise comparisons to probe-level data [16-19]. On average, the Affymetrix 3' IVT platform contains 22 probes for every transcript represented on the array. Using this information directly, the S-Score algorithm has shown good sensitivity when compared to many other existing analysis methods without sacrificing specificity (including RMA, dChip and MAS5), and can produce accurate results when no biological replicates are present [18,19]. This is particularly applicable and appropriate to our individual time point datasets in which we have only one paired array set for each time point. Using the S-Score algorithm, the relative change in probe pair intensity is calculated to convert the probe pair signal differences into multiple measurements with equalized errors. The relative changes for each probe pair are then summed to form the S-score, which represents a single measure of the significance of change for the gene in question[19]. By definition, S-score is related to *P*-value by an exponential relation, and a value of 3 corresponds to a *P*-value of 0.003 [16,19,19]. Genes with an S-score ≥ 3.0 or ≤ - 3.0 (*P *≤ 0.003) were selected for further analysis.

### Ingenuity Pathways Analysis

For selected genes (genes with a *P*-value < 0.001 and a fold-change ≥ 1.5 or ≤ -1.5 for the time-independent analysis; genes with an S-score ≥ 3.0 or ≤ - 3.0 (*P *≤ 0.003) for the time-dependent analysis), Ingenuity Pathway Analysis (IPA) version 2.0 (Ingenuity^® ^Systems Inc, Redwood City, CA; http://www.ingenuity.com) was used to search for biological functions and interrelationships between significantly modulated genes in PNY versus SHAM mice. IPA provides a large manually curated database containing over 200,000 full text articles and information about thousands of human, mouse and rat genes [20] with which experimental data sets can be statistically compared. Genes from the dataset were overlaid onto a global molecular network developed from information contained within the IPA database, and networks of genes in the dataset were then algorithmically generated based on their connectivity (both direct and indirect relationships). Each network displays the type of relationship between two gene products, including genes that are not significantly altered in the user's microarray data set. The networks are ranked depending on the number of significantly expressed genes they contain, based on a *P*-value that indicates the likelihood of the genes in a network being found together due to chance. A score of 2 indicates a 1 in 100 chance that the focus genes of interest were linked in the network by chance rather than a direct biological relationship. Therefore, scores of 2 or higher have at least a 99% confidence level of not being generated by random chance alone [20].

### Quantitative reverse transcription PCR validation

Total RNA from individual lung tissue samples (n = 3) from each group (PNY vs. SHAM) at the 1 day time point was prepared using TRIzol (Invitrogen, Carlsbad CA) as recommended by the manufacturers, followed by the Qiagen RNAeasy mini kit (Qiagen #74104) according to the manufacturer's directions. Total RNA concentrations and RNA quality was determined using an Agilent Bioanalyzer (Agilent Technologies Inc, Wilmington DE), with RIN > 7 for all samples. The RNA from each of the six individual samples was then subjected to genomic DNA elimination and first strand cDNA synthesis using a commercial kit (RT^2 ^First Strand Kit, SA Biosciences) to generate the cDNA templates for PCR amplification. Quality control was performed using the SA Biosciences QC qRT-PCR array (SA Biosciences) to test for any inhibition of cDNA synthesis, or presence of genomic DNA contamination. Gene expression assays were performed using sets of premade mouse primer pairs (SA Biosciences) for Igf1, Cyr61, Igfbp2, Igfbp3, Tnfrsf12a, Tnc, Col3A1 and Eln (see Table 1). Quantitative PCR was performed using a Stratagene MX3000P Detection system, and RT^2 ^qPCR SYBR green PCR Master Mix (SA Biosciences), according to the manufacturer's recommended protocol. Each sample was analyzed in triplicate, and relative gene expression (PNY versus SHAM) was calculated using the comparative Ct method [21] after normalization to the housekeeping gene Gapdh, which did not show differences in expression between SHAM and PNY mice (see online microarray dataset - accession number GSE15999 at: http://www.ncbi.nlm.nih.gov/geo/query/acc.cgi?acc=GSE15999).

**Table 1:** Validation of the microarray data using quantitative rt-PCR

Pathway	Gene	SABiosciences Catalog #	q-rtPCR (d1)*	Microarray (TI)*
				

Igf-1 signaling	Igf1	PPM03387E	1.4 (1.0 - 1.9)	1.5
	Cyr61	PPM05012A	4.0 (3.0 - 4.9)	1.6
	Igfbp2	PPM05178A	1.1 (-1.6 - 2.0)	1.5
	Igfbp3	PPM03820E	-4.0 (-8.0 - -4.0)	-1.6
Fibroblast activation	Tnfrs12a	PPM27298A	2.8 (1.8 - 4.3)	1.5
	Tnc	PPM03804E	5.0 (2.6 - 9.8)	2.2
	Col3A1	PPM04784B	1.2 (-1.1 - 1.6)	1.5
	Eln	PPM36834A	1.4 (1.1 - 2.2)	1.6

* The numbers represent the mean fold change for each gene transcript. The numbers in brackets represent the estimated range of fold change of gene expression seen in pneumonectomy animals (n = 3) compared to sham animals (n = 3), based on the standard error calculated from the pneumonectomy ΔCt values. For the microarray data, all expression values are significant with *P *< 0.001. TI - time-independent.

### Animals used for immunohistochemical study

Mice used for the immunohistochemistry study were also adult (10-12 week) female C57BL/6 (20-25 g) obtained from Jackson Laboratories. For each time point (3 days and 7 days), the mice were divided into two groups: (1) pneumonectomy (PNY) and (2) sham operated (SHAM - thoracotomy without lung resection), with three animals in each group. Unilateral pneumonectomy or sham thoracotomy were performed as described above, and after recovery, all mice were fed BrdU in drinking water (0.8 mg/ml) between days 0-3 (day 3 time point) or 4-7 (day 7 time point) before euthanasia.

### Immunohistochemistry

The mice were anesthetized as above at 3 days and 7 days after surgery (PNY or SHAM) then euthanized by cervical dislocation. Following median sternotomy, the pulmonary vasculature was perfused with ice cold Hanks balanced salt solution, the trachea cannulated, and the lungs removed *en bloc*. Tissue fixation was achieved with intratracheal 10% buffered formalin at 25 cmH_2_0 overnight. The trachea was then ligated, and the lung was embedded in paraffin. Immunofluorescent staining (IF) was performed on 5 μm paraffin sections. Primary antibodies included the monoclonal mouse antibody anti-BrdU (Santa Cruz, dilution 1:100), the monoclonal rabbit antibody anti-S100A4 (AbCam, dilution 1:100), and the monoclonal mouse antibody anti-αSMA (Santa Cruz, dilution 1:100). Tissue sections were deparaffinized and hydrated using standard methods, and antigen retrieval was performed using a citrate buffer (pH 6.0) and microwave heating (5 mins at high, 15 mins at 40% power). Tissues were washed (TBS with 0.1% Tween) three times before a 20 minute protein block (Dako, Carpinteria, CA), and then exposed to the primary antibodies (15-18 hours at 4 degrees Celsius). Detection of the primary antibodies was achieved using donkey anti-mouse Alexa Fluor 594 (red) for BrdU and donkey anti-mouse or donkey anti-rabbit Alexa Fluor 488 (green) for αSMA and S100A4 respectively, both at 1:200 (30 mins at 37 degrees Celsius). The appropriate isotype control assays were also performed; non-specific staining was not observed.

To examine the proliferation of S100A4 positive parenchymal cells during lung regeneration, 20 randomly selected high power fields (400×) were photographed digitally for each sample (3 PNY and 3 SHAM animals at each time point). Cells were counted (averaging 50-100 nucleated cells/HPF) and divided into four categories (nucleated cells (DAPI), S100A4 positive, BrdU positive, and double positive (S100A4+BrdU), and the mean percentage of S100A4 cells/nucleated cells, BrdU cells/nucleated cells and S100A4+BrdU/nucleated cells were obtained. A two-way ANOVA and independent t-tests were performed to test for significance (*P *< 0.05) between PNY and SHAM, and between 3 day and 7 day time points.

## Results

### Microarray analysis and validation

Microarray data was collected from pooled lung samples (n = 8) at four time points (6 hr, 1 day, 3 day and 7 day) during post-pneumonectomy lung regeneration from both PNY and SHAM animals. As mentioned in the methods section, the data obtained from these microarrays were analyzed in two different ways. First, data from each time point was combined in a non-parametric replicate analysis generating a time-independent data set, PNY vs. SHAM, with four replicates. In this time-independent analysis to identify consistently regulated genes, 179 genes were identified as differentially expressed between PNY and SHAM (*P *< 0.001) with fold changes of ≥ 1.5 or ≤ -1.5 (Table S1, additional file 1). Second, global gene expression patterns in the lung were analyzed independently at each of the four time points following pneumonectomy (6 hours, 1 day, 3 day and 7 day). In this time-dependent analysis, 346, 472, 556 and 733 genes were differentially expressed between PNY and SHAM at 6 hour, 1 day, 3 day and 7 day post-pneumonectomy respectively (complete data not shown), with an S-score of ≥ 3 or ≤ -3 (equivalent to *P *< 0.003). In addition, validation of the microarray data was performed using quantitative rt-PCR. PCR was performed using 8 genes that showed modulated expression across several different areas of interest at the 1 day time point, as well as in the time-independent analysis. The expression patterns observed using qRT-PCR are similar to patterns observed by microarray (see Table 1).

### Time-independent transcriptomic patterns during lung regeneration

A complete list of genes with significantly (*P *< 0.001) altered expression (fold change ≥ 1.5, or ≤ -1.5) in the time-independent analysis of post-pneumonectomy lung regeneration is compiled in Table S1, additional file 1. This table is organized into biological functions that are prevalent during lung regeneration including cell cycle/cell division, DNA synthesis or repair, cell proliferation, extracellular matrix, cytoskeleton, inflammatory, fibrotic, and immune responses, and protein phosphorylation, and miscellaneous biological functions. From this table, several important transcriptomic patterns emerge. First is the significant up-regulation of many cell cycle, cell division, and DNA synthesis-related genes. Many genes involved in cell proliferation are also differentially expressed, including members of Igf1 signaling (up-regulation of Igf1, Igfbp2, Cyr61 and Pappa2 and down-regulation of Igfbp3), as well as Ctgf, Hbegf and Tnfrsf12a. Components of the extracellular matrix including Tnc, Eln, Fbn1, Col3A1, Col5a2 and Vcan are up-regulated, as well as two members of the Adamts metalloproteinase family (Adamts2, Adamts9). Interestingly, genes relating to goblet cell hyperplasia and mucous production are also up-regulated (Clca3, Agr2, Slc26a4), with differential expression of other genes associated with inflammatory, fibrotic or immune responses (up-regulation of Reg3g, Ear11, Retnla, Nappa, and Ccr9; down-regulation of Arg1, CD5L and Alox15).

**Figure 1:**
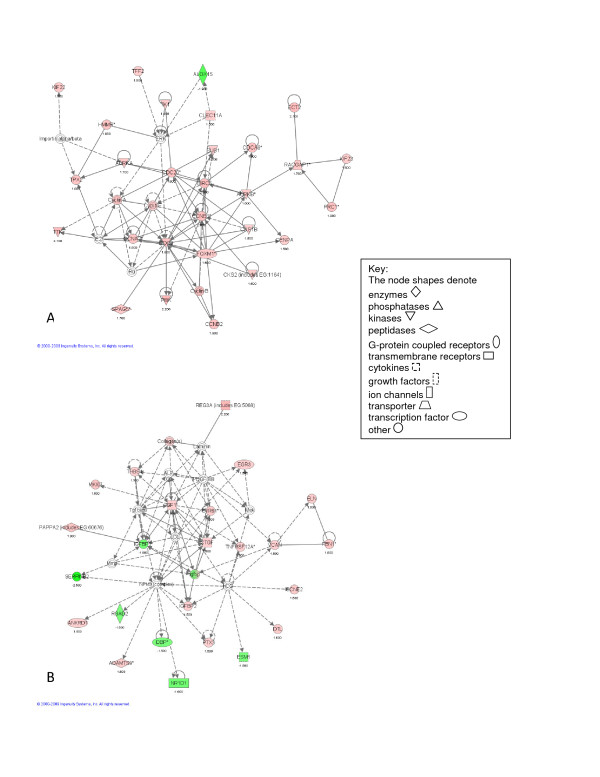
**Illustrations of the top gene networks for the time-independent microarray analysis**. A - Most significant network for the time-independent microarray analysis (score = 56). B - Second most significant network for the time-independent microarray analysis (score = 45).

Figure 1 illustrates the top networks defined by IPA for the time-independent analysis of post-pneumonectomy lung regeneration. These networks represent diverse relationships (represented as a line) between different genes (represented as filled shapes). Red nodes represent genes that *increased *in expression in animals after pneumonectomy compared to sham-operated animals, whereas green nodes represent genes that *decreased *in expression in animals after pneumonectomy compared to sham-operated animals. IPA network analysis corroborated the importance of cell cycle regulation, cell movement and cell proliferation during lung regeneration (Figure 1A and 1B), with the top two most significant networks focused on cell cycle (Ccnbl, Cc2, Ccna2, Birc5 and Foxm1), and mesenchymal cell proliferation (Igf1, Cyr61, Tnfrsf12a, Ctgf, Igfbp3 and Ifgbp2) respectively.

### Time-dependent transcriptomic patterns during lung regeneration

**Figure 2:**
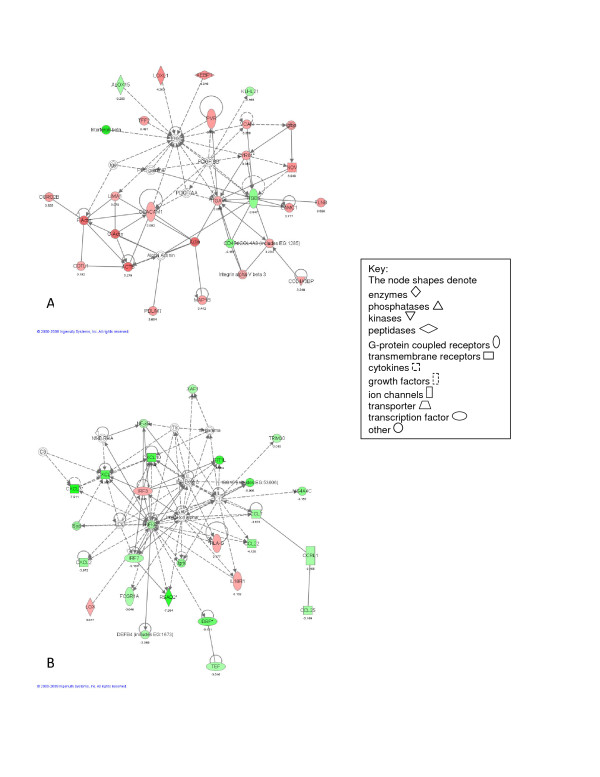
**Illustrations of the top gene networks at the 6 hour time point of the time-dependent microarray analysis**. A - most significant gene network (score = 38). B - second most significant network (score = 36).

**Figure 3:**
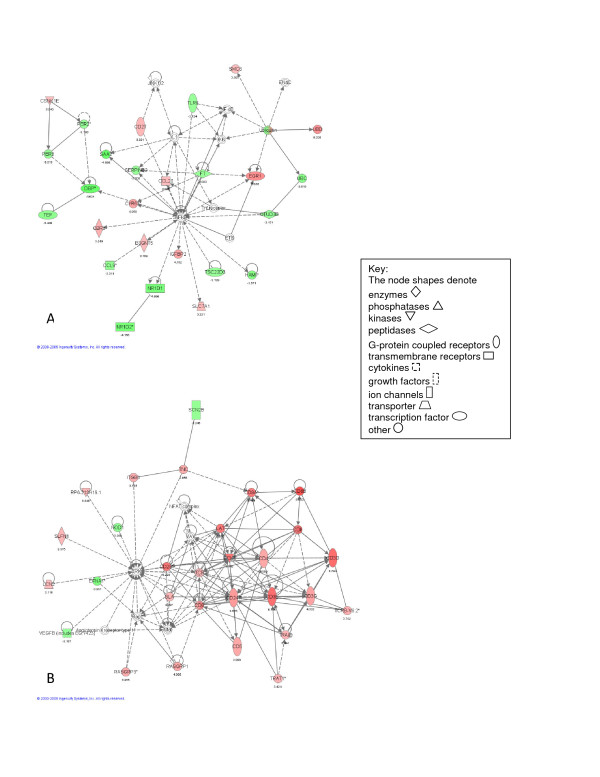
**Illustrations of the top gene networks at the 1 day time point of the time-dependent microarray analysis**. A - most significant gene network (score = 42). B - second most significant network (score = 38).

**Figure 4:**
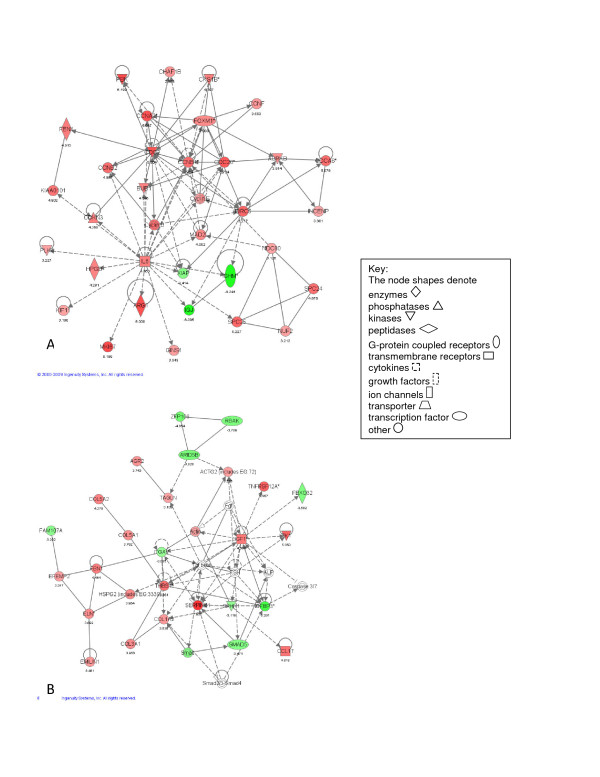
**Illustrations of the top gene networks at the 3 day time point of the time-dependent microarray analysis**. A - most significant gene network (score = 57). B - second most significant network (score = 45).

**Figure 5:**
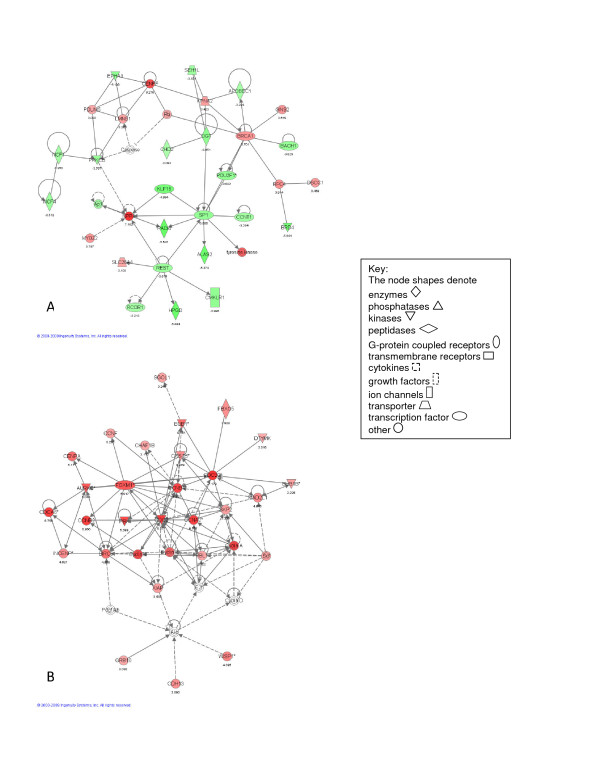
**Illustrations of the top gene networks at the 7 day time point of the time-dependent microarray analysis**. A - most significant gene network (score = 48). B - second most significant network (score = 42).

The analysis of differentially expressed transcripts observed at each of the four individual time points is summarized by the top networks (Figures 2, 3, 4 and 5) as defined by IPA. As demonstrated in Figure 2, the top networks identified by IPA at 6 hours after pneumonectomy are associated with cell-cell signaling (including up-regulation of Ceacam1 and ItgaV - Figure 2A), and inhibition of inflammatory cell migration (down-regulation of the pro-inflammatory cytokines Cxcl1 and Cxcl10, as well as Nfκβ - Figure 2B), demonstrating a major shift in regulation of cell adhesion and inflammatory response at this early time point. The top networks identified by IPA at 1 day (Figure 3) are associated with continued modulation of the inflammatory response (up-regulation of Egr1, but down-regulation of Dbp and SerpinB2 - Figure 3A), especially focused on T-cell development and function (up-regulation of Cd4, Cd3e, Cd28, Cd247 and Lak - Figure 3B). At 3 days the top networks identified by IPA involve cell cycle progression (up-regulation of Foxm1, Cdc2, Cdc20, Ccnd1 and IL6 - Figure 4A), and activation of fibroblastic cells with production of extracellular matrix components (up-regulation of Igf1, Tnfrsf12A, Eln, Fbn1, Col3A1, Serpine1 and Thbs1 - Figure 4B). Finally, at 7 days, the top networks are focused on immunomodulation and inflammatory responses (up-regulation of NPPA/ANP and Brca1 - Figure 5A), and continued cell proliferation (up-regulation of Birc5, Foxm1, Cdc2, Ccnb1 and Ccna2 - Figure 5B).

### Proliferation of S100A4 positive parenchymal cells following pneumonectomy

**Figure 6:**
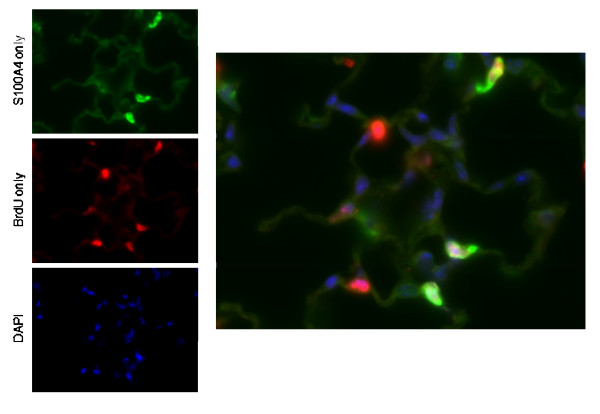
**Topography of S100A4/BrdU double positive alveolar cells**. Photomicrographs (630× magnification) of double positive staining cells, with S100A4 (green), BrdU (red) and nuclei (blue).

**Figure 7:**
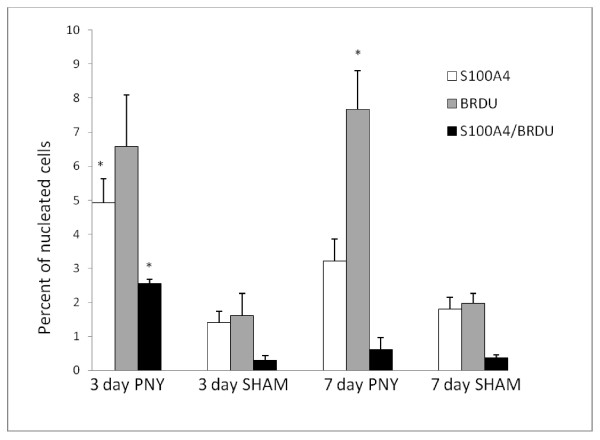
**Percentage of nucleated cells that stained positive for either S100A4, BrdU or double positive for S100A4 and BrdU**. Data are mean values ± SE. *P < 0.05 between PNY and SHAM for a given time point.

**Figure 8:**
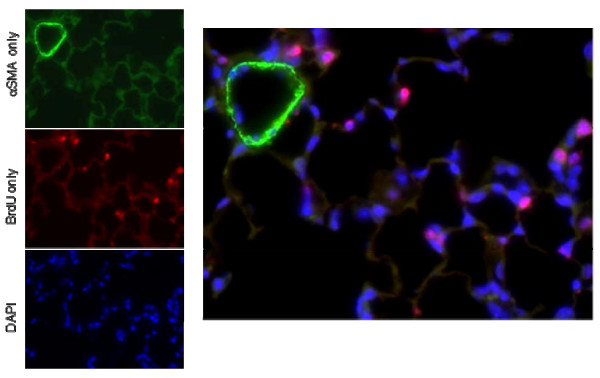
**Topography of αSMA positive cells 7 days after pneumonectomy**. Photomicrograph (400× magnification) with αSMA (green), BrdU (red) and nuclei (blue), illustrating αSMA staining around a vessel, but not in surrounding alveoli or associated with BrdU positive cells. Similar results were seen at 3 days after pneumonectomy and in sham-operated animals (results not shown).

Analysis of transcriptomic patterns during post-pneumonectomy lung regeneration suggests an integral role for mesenchymal cells, in particular in the production of extracellular matrix as mentioned above. To further elucidate the role of mesenchymal cells, an immunohistochemical analysis of the regenerating parenchyma was performed using the fibroblast marker S100A4 (Fsp1) [22-24]. Mice were fed BrdU in drinking water between days 1-3 (measured on day 3) or on days 4-7 (measured on day 7) post-pneumonectomy or sham thoracotomy. To understand their role in alveologenesis, S100A4+ cells were enumerated specifically in the alveolar parenchyma (Figure 6), although proliferating S100A4+ cells were also seen in the perivascular, peribronchiolar, and pleural regions. After PNY, there was a significant (*P *< 0.05) increase in the percentage of total nucleated cells that were positive for S100A4, BrdU, or S100A4 in combination with BrdU (Figure 7). Significantly more S100A4+ or S100A4+/BrdU+ cells were observed after PNY (vs SHAM) on day 3 but not on day 7, although the percentage of BrdU+ cells was increased to similar levels by PNY on days 3 and 7. Therefore, it appears that S100A4+ cells proliferated only in the early period of lung regeneration (day 1-3). In contrast to S100A4, the myofibroblast marker αSMA was only detected in the perivascular and peribronchial regions, and these cells were not associated with BrdU uptake (Figure 8).

## Discussion

The murine response to pneumonectomy represents a unique biological phenomenon among adult mammals. By understanding the molecular signaling that defines alveolar regeneration in this species, novel targets to promote lung regeneration in higher mammals may be revealed. Transcriptomic patterns and cell proliferation data during post-pneumonectomy lung regeneration suggest that mesenchymal cells appear to be critical for rebuilding the infrastructure of the lung in this setting. Their proliferation corresponds to an increase in expression of extracellular matrix structural genes and proteases that are essential for tissue remodeling. While it may seem obvious that mesenchymal cells rebuild the post-pneumonectomy lung, the specific pattern of transcription and associated gene networks have not been systematically evaluated in this model. Patterns of growth factor expression suggest that these cells influence the growth and migration of epithelial and endothelial cells in a paracrine fashion. Furthermore there is an increase in expression of "anti-inflammatory" cytokines which may be important in preventing an over-exuberant innate immune response, and for prevention of fibrosis. Which of these processes are specifically carried out by mesenchymal cells warrants further investigation. However, our initial analysis shows that S100A4+ fibroblast-like cells, but not αSMA+ myofibroblasts, are proliferating cells that contribute to the transcriptomic pattern observed here.

### Mesenchymal cell proliferation and immunomodulation during lung regeneration

The transcriptomic patterns suggest that mesenchymal cells are activated during lung regeneration, resulting in increased expression of common extracellular matrix components, in concert with transcriptional control of inflammatory and immunomodulatory pathways. The end result of this well orchestrated process is a non-fibrogenic form of 'wound healing'. The role of fibroblasts and myofibroblasts in alveolar regeneration was further investigated using immunohistochemical analysis of two markers, S100A4 and αSMA that have been previously associated with fibroblasts and myofibroblasts respectively [22-24]. This analysis confirmed that S100A4-positive fibroblastic cells proliferate in the lung parenchyma during lung regeneration (3 days), but are later down-regulated (7 days). Although S100A4 was not up-regulated at the transcriptional level during lung regeneration, this is consistent with regulation via post-transcriptional modulation as seen in other systems [25]. In contrast, αSMA staining was only associated with vessels and not proliferating parenchymal cells, suggesting that myofibroblasts are not highly active in the process of alveolar regeneration. These data suggest that while fibroblast proliferation is important during lung regeneration, subsequent down-regulation of fibroblast proliferation and minimization of myofibroblastic differentiation may be equally important to avoid parenchymal fibrosis.

Persistent proliferation of S100A4-positive fibroblasts has been associated with fibrotic diseases such as murine bleomycin-induced fibrosis, in which S100A4-positive cell numbers peak at 2-3 weeks and are still above baseline at 4 weeks [23]. Therefore, it is crucial to better understand how fibroblast proliferation can participate in lung regeneration without myofibroblastic differentiation and/or subsequent fibrosis. One possibility is that a combination of reduced Tgfβ signaling activity and reduced pro-inflammatory Th-2 cytokine production controls this response. For example, atrial natriuretic peptide (ANP, encoded by Nappa) is up-regulated during post-pneumonectomy lung regeneration, but down-regulated in OVA-induced asthma, a process associated with a fibrotic response [26]. ANP is anti-fibrotic, and acts through inhibition of Tgfβ-induced fibroblast transformation [27]. The combined effects of different growth factors can also modulate the actions of Tgfβ on cell proliferation and collagen production in fibroblast trans-differentiation. For example, Tgfβ generally induces myofibroblast differentiation, with concurrent increased production of αSMA and type I and III collagen [28]. However, the presence of either Egf or Igf can influence fibroblasts to undergo cell proliferation and DNA synthesis (Igf), or differentiation and αSMA production (Egf) [28]. The actions of Igf1 depend largely on its binding to extracellular proteins such as Igfbp2 and Igfbp3 [29-31]. While Igf1 and Igfbp2 are up-regulated during lung regeneration, Igfbp3 is down-regulated. Increased Igfbp3 expression is associated with emphysema [32] and senescent fibroblasts, where it results in Igf1 sequestration and reduced cell proliferation [33,34]. Conversely, Igfbp2 binds to extracellular matrix or fibroblasts in the presence of Igf1 or Igf2 and increases their local bioavailability [35,36]. Since Igf1 signaling is highly invoked during post-pneumonectomy lung regeneration, the actions of Igf1 might contribute to a proliferative rather than fibrotic fibroblast phenotype.

Although fibroblast proliferation is a common feature of lung regeneration and pulmonary fibrotic diseases, the early (6 hour and 1 day) down-regulation of cytokines and chemokines involved in inflammatory cell migration (Figure 2B), and up-regulation of genes involved in T cell development and function (Figure 3B) is unique to lung regeneration. Arginase 1 (Arg1) expressed by Th2-induced fibroproliferative M2 macrophages [37] is also up-regulated during OVA-induced asthma [26], but down-regulated during post-pneumonectomy lung regeneration. These data suggest an important role for reduced inflammatory response, immunomodulation, and M1 rather than M2 macrophages in successful lung repair/regeneration. These patterns may represent important mechanisms for balancing cell proliferation and extracellular matrix remodeling without incurring fibrotic scarring resulting from the action of exuberant pro-inflammatory cascades. In contrast to the early anti-inflammatory transcriptomic pattern observed during lung regeneration in this study, another recent study describes up-regulation of select inflammatory cytokines (Hmgb1 and INF-y) during post-pneumonectomy lung regeneration, although most cytokines studied were unaffected by the procedure [38].

### Does adult alveolar regeneration recapitulate lung alveolar development?

Both the observed transcriptomic patterns and the results of the immunohistochemical analysis suggest that adult alveolar regeneration is not identical to lung alveolar development. Myofibroblasts have been previously described as essential effector cells in post-natal secondary septation and alveolarization, with characteristic α-SMA synthesis and localization to developing alveolar septa [39,40]. In contrast, little αSMA expression is present in the parenchyma during post-pneumonectomy lung regeneration, and immunohistochemical localization of αSMA suggests that activated myofibroblasts are absent from alveolar structures. Igf signaling and mRNA expression patterns are up-regulated during alveolar development [41,42], a trend which is also apparent in lung regeneration transcriptomic patterns. However, other common signaling pathways such as Fgf [43-45], Pdgf signaling [46] and HoxA5 [47] have been implicated in alveolar development, but are not significantly present in transcriptomic patterns during lung regeneration. These differences could represent alterations between the ontogeny of alveolar development and post-pneumonectomy lung regeneration, or may reflect the broad experimental design of our study, which may have reduced our ability to detect subtle and/or temporally restricted changes.

## Conclusion

Analysis of transcriptomic patterns at four time points during post-pneumonectomy lung regeneration reveals significant insight into the regeneration of normal lung tissue after partial pneumonectomy. By including both time-independent and time-dependent analyzes, the data provides insight into important checks and balances that are activated to facilitate growth of functional lung tissue without fibrogenesis. Analysis of the data set using IPA revealed two themes that are important in the process of lung regeneration. The first is the transcriptomic patterns consistent with activation of mesenchymal cells, and the second is transcriptomic patterns consistent with anti-inflammatory immunomodulatory activity. The presence of proliferating mesenchymal cells in the alveolar parenchyma was also demonstrated immunohistochemically. Although proliferating S100A4+ cells (fibroblasts) have been previously associated with fibrotic scarring, this data demonstrates that S100A4+ cells can actively participate in non-fibrogenic tissue regeneration in the lung [48]. In lung regeneration, it may be the influence of immune modulation and modulation of the inflammatory response that is responsible for balancing cell proliferation and extracellular re-modeling against fibrosis.

Taken together with previous reports examining the mechanism of lung regeneration, we can speculate that from the initial stimulus of increased mechanical stress and hypoxia [2], post-pneumonectomy lung regeneration occurs through a combination of early immediate gene expression [9,10], up-regulation of genes important in cell cycle regulation and cell proliferation, and the careful orchestration of fibroblast proliferation, extracellular matrix deposition and immunomodulation to prevent excessive fibrosis. Our study provides a unique description of transcriptomic patterns and mesenchymal cell proliferation during post-pneumonectomy lung regeneration in the adult mouse. However, better understanding of biologically important mechanisms using transgenic models, lineage tagging, and transplant models will be important to further understand the process of lung regeneration.

## Competing interests

The authors declare that they have no competing interests.

## Authors' contributions

JAP participated in the design of the study, carried out the RNA preparation, PCR, immunohistochemistry, data analysis and manuscript preparation. CDP and LKI performed the statistical analyzes. MRM participated in the study design and immunohistochemistry. EPI participated in study design, data review and manuscript review. AMH conceived of the study, participated in the study design and in drafting the manuscript. All authors read and approved the final manuscript.

## Supplementary Material

Additional file 1 **Table S1 - Transcripts with significant (*P *< 0.001) differential expression (PNY vs SHAM) during lung regeneration (time-independent analysis)**. This table provides a categorized list of all transcripts showing differential expression (PNY vs SHAM) by microarray as identified through time-independent analysis. Click here for file
