# Supplementary material for: Global gene expression patterns in the post-pneumonectomy lung of adult mice
Source: Respir Res. 2009 Oct 5;10(1):92. doi: 10.1186/1465-9921-10-92 (PMC2770038; doi:10.1186/1465-9921-10-92)
Supplement: Additional file 1 — Table S1 - Transcripts with significant (P < 0.001) differential expression (PNY vs SHAM) during lung regeneration (time-independent analysis). This table provides a categorized list of all transcripts showing differential expression (PNY vs SHAM) by microarray as identified through time-independent analysis. [file 1465-9921-10-92-S1.DOC]

Table S1 – Transcripts with significant (*P* < 0.001) differential expression (PNY vs SHAM) during lung regeneration (time-independent analysis)

| **Gene** | **FC** | **Description** |
| --- | --- | --- |
| *Cell cycle/cell division*  Aspm  Ect2  Esco2  Ccnb1-rs1  Cep55  Nuf2  Birc5  Cenpf  Prc1  Cdc20  Ccne2  Ccnb2  Cdca8  Ube2c  Mki67  Tpx2  Nusap1  Ncapg  Ccna2  Spbc25  Cenpi  Cenpp  Aurka  Spag5  Stil  Racgap1  Kif20a  Shcbp1  Chek1  Cdca5  Cdca2  Ncapd2  Cdca3  Plk1  Cks1b  Cks2  Sgol2  Spbc24  Cenpk  Cenpa  Zwilch  Kntc1 | 2.4  2.1  2.1  2.0  2.0  2.0  2.0  1.9  1.9  1.9  1.9  1.9  1.9  1.8  1.8  1.8  1.8  1.8  1.8  1.8  1.7  1.7  1.7  1.7  1.7  1.7  1.6  1.6  1.6  1.6  1.6  1.6  1.5  1.5  1.5  1.5  1.5  1.5  1.5  1.5  1.5  1.5 | Asp(abnormal spindle)-like, microcephaly associated)  Ect2 oncogene  Establishment of cohesion 1 homolog 2 (S.cerevisiae)  cyclin B1, related sequence 1  Centrosomal protein 55  NUF2, NDC80 kintochore complex component  Baculoviral IAP repeat-containing 5  Centromere protein 2  Protein regulator of cytokinesis 1  Cell division cycle 20 homolog (S.cerevisiae)  cyclin E2  Cyclin B2  Cell division cycle associated 8  Ubiquitin-conjugating enzyme E2C  Antigen identified by monoclonal antibody Ki67  Tpx2, microtubule-associated protein homolog  nuclear and spindle associated protein 1  Non-SMC condensing 1 complex, subunit G  Cyclin A2  Spindle pole body component 25 homolog (S.cerevisiae)  Centromere protein 1  Centromere protein P  Aurora kinase A  Sperm associated antigen 5  Scl/Tal1 interrupting locus  Rac GTPase-activating protein 1  Kinesin family member 20A  Shc SH2-domain binding protein 1  Checkpoint kinase 1 homolog (S.pombe)  Cell division cycle associated 5  Cell division cycle associated 2  Non-SMC condensing 1 complex, subunit D2  Cell division cycle associated 3  Polo-like kinase 1 (Drosophila)  CDC28 protein kinase 1b  CDC28 protein kinase regulatory subunit 2  Shugoshin-like 2 (S.pombe)  Spindle pole body component 24 homolog (S.cerevisiae)  Centromere protein K  Centromere protein A  Zwilch, kinetochore associated, homolog (Drosophila)  Kinetochore associated 1 |
| *DNA synthesis and repair*  Top2a  Rrm2  Exo1  Rad51ap1  Tk1  Dtl  Rad51  Smc2  Brca1  Rfc4  Uhrf1 | 1.9  1.8  1.7  1.7  1.6  1.6  1.6  1.6  1.5  1.5  1.5 | Topoisomerase (DNA) II alpha  Ribonucleotide reductase M2  Exonuclease 1  RAD51 associated protein 1  Thymidine kinase 1  Denticleless homolog (Drosophila)  RAD51 homolog (S.cervisiae)  Structural maintenance of chromosomes 2  Breast cancer 1  Replication factor C (activator 1) 4  Ubiquitin-like, containing PHD and RING finger domains, 1 |
| *Cell proliferation*  Pappa2  Areg  Iqgap3  S100a14  Psat1  Fignl1  Hells  Foxm1  Wisp1  Cyr61  Ncapg2  Tnfrsf12a  Igf1  Ctgf  Ankrd1  Ifgbp2  Clec11a/SCGF  Hbegf  Edg8  Esm1  Chrdl1  Dbp  Bach1  Klf15  Igfbp3  Serpinb2/PAI-2 | 1.8  1.8  1.7  1.7  1.7  1.6  1.6  1.6  1.6  1.6  1.5  1.5  1.5  1.5  1.5  1.5  1.5  1.5  -1.5  -1.5  -1.5  -1.5  -1.5  -1.5  -1.6  -2.5 | Pappalysin 2 - cleaves Igfbps  Amphiregulin  IQ motif containing GTPase activating protein 3  S100 calcium binding protein A14  Phosphoserine aminotransferase 1  Fidgetin-like 1  Helicase, lymphoid specific  Forkhead box M1  WNT1 inducible signaling pathway protein 1  Cysteine rich protein 61  Non-SMC condensin II complex, subunit G2  Tumor necrosis factor receptor superfamily, member 12a  Insulin-like growth factor 1  Connective tissue growth factor  Ankyrin repeat domain 1 (cardiac muscle)  Insulin-like growth factor binding protein 2  C-type lectin domain family 11, member a/stem cell growth factor  Heparin-binding EGF-like growth factor  Endothelial differentiation, sphingolipid Gprotein-coupled receptor, 8  Endothelial cell-specific molecule 1  Chordin-like 1  D site albumin promoter binding protein  BTB and CNC homology 1  Kruppel-like factor 15  Insulin-like growth factor binding protein 3  Serine (or cysteine) peptidase inhibitor, cladeB, member 2 |
| *Extracellular matrix*  Tnc  Chi3l4  Fbn1  Adamts9  Eln  Mest  Adamts2  Col3A1  Cilp  Vcan  Col5a2  Agrin  Prg4 | 2.2  1.9  1.8  1.6  1.6  1.6  1.6  1.5  1.5  1.5  1.5  -1.5  -1.7 | Tenescin C  Chitinase 3-like 4/Ym2  Fibrillin 1  ADAM, thrombospondin type 1, motif 9  Elastin  Mesoderm specific transcript  ADAM, tps type 1, motif 2  Procollagen, type III, alpha 1  Cartilage intermediate layer protein  Versican  Procollagen, type V, alpha 2  Agrin  Proteoglycan 4 (megakaryocyte stimulating factor) |
| *Cytoskeleton*  Kif11  Kif22  Myl1  Actg2  Kif23  Mphosph1  Cttnbp2  Ckap2  Eml4  Fbox32 | 1.9  1.7  1.6  1.6  1.5  1.5  1.5  1.5  1.5  -1.5 | Kinesin family member 11  Kinesin family member 22  Myosin, light polypeptide 1  Actin, gamma 2, smooth muscle, enteric  Kinesin family member 23  M-phase phosphoprotein 1  Contactin binding protein 2  Cytoskeleton associated protein 2  Echinoderm microtubule associated protein like 4  F-box protein 32 |
| *Inflammatory/*  *fibrotic and immune response*  Clca3  Reg3g  Ear11  Retnla  Slc26a4  Nappa  Agr2  Thbs1  Egr3  Igh-6  Ptx3  Tff2  Orm1  Ramp2  Nr1d1  Arg1  CD5l  Zbtb16  Alox15 | 6.5  2.6  1.8  1.7  1.7  1.7  1.6  1.5  1.5  1.5  1.5  1.5  1.5  -1.5  -1.6  -1.6  -1.7  -1.7  -1.7 | Chloride channel calcium activated 3  Regenerating islet-derived 3  Eosinophil-associated, ribonuclease A, member 11  Resistin like alpha/FIZZ1  Solute carrier family 26, member 4  Natriuretic peptide precursor type A (ANP)  Anterior gradient 2 (Xenopus laevis)  Thrombospondin 1  Early growth response 3  Immunoglobulin heavy chain 6 (heavy chain of IgM)  Pentraxin related gene  Trefoil factor 2 (spasmolytic protein 1)  Orosomucoid 1  Receptor (calcitonin) activity modifying protein 2  Nuclear receptor subfamily 1, group D, member 1  Arginase1, liver  CD5 antigen-like  Zinc finger and BTB domain containing 16  Arachidonate 15-lipoxygenase |
| *Protein phosphorylation*  Pbk  Ttk  Cdkn3  Ube2t  Dusp8  Melk  Bub1  Cdkl4 | 2.2  2.1  1.9  1.7  1.7  1.6  1.5  -1.5 | PDZ binding kinase  Ttk protein kinase  Cyclin-dependent kinase inhibitor 3  Ubiquitin-conjugating enzyme E2T (putative)  Dual specificity phosphatase 8  Maternal embryonic leucine zipper kinase  Budding uninhibited by benzimidazoles 1 homolog  Cyclin-dependent kinase-like 4 |
| *Misc*  Depdc1a  C79407  Hmmr  Dio2  Syn2  Trip13  D2Ertd750e  Ppil5  Car3  Cyp51  Slitrk6  Tcf19  Phex  Srl  Sprr1a  Trim59  Pla1a  Trps1  Rsad2  Galntl2  Samd12  Fnip1  Klhl24  Snca  Alas2  Hba-a1 | 2.3  2.0  1.8  1.8  1.7  1.7  1.7  1.6  1.6  1.5  1.5  1.5  1.5  1.5  1.5  1.5  1.5  1.5  -1.5  -1.5  -1.5  -1.5  -1.6  -1.6  -1.9  -1.9 | DEP domain containing 1a  Expressed sequence C79407  Hyaluronan mediated motility receptor (RHAMM)  Deiodinase, iodothyronine, type II  Synapsin II  Thyroid hormone receptor interactor 13  DNA segment, Chr 2, ERATO Dio 750, expressed  Peptidylprolyl isomerase (cyclophilin) like 5  Carbonic anhydrase 3  Cytochrome P450, family 51  SLIT and NTRK-like family, member 6  Transcription factor 19  Phosphate regulating gene with homology to endopeptidases on X Sarcalumenin  Small proline-rich protein 1A  Tripartite motif-containing 59  Phospholipase A1 member A  Trichorhinophalangeal syndrome I  Radical S-adenosyl methionine domain containing 2  UDP-N-acetyl-alpha-D-galactosamine  Sterile alpha motif domain containing 12  Folliculin interacting protein 1  Kelch-like 24 (Drosophila)  Synuclein, alpha  Aminolevulinic acid synthase 2, erythroid  Hemoglobin alpha, adult chain 1 |
